# The role of newspapers published in North Sumatra during Indonesia's independence struggle between 1916-1925: A discourse analysis

**DOI:** 10.12688/f1000research.53442.1

**Published:** 2022-02-28

**Authors:** Ichwan Azhari, Ricu Sidiq, Ika Purnamasari

**Affiliations:** 1 Department of Historical Education, Universitas Negeri Medan, Medan, Sumatera Utara, 20225, Indonesia

**Keywords:** Newspapers, nationality, independence, North Sumatra

## Abstract

**Background:** In the history of Indonesian independence, newspapers not only functioned as a medium of information but also as a tool for the struggle for independence. This is because of the role of newspapers in bringing out the spirit of nationalism and the concept of “nation” to the Indonesian people through various writings and reports in each edition. This study aims to describe the form of Indonesia's struggle for independence in newspapers published in North Sumatra in 1916-1925.

**Methods: **This research uses the critical discourse analysis method. The research data are in the form of texts published in thirteen indigenous newspapers published in North Sumatra in 1916-1925. Data analysis used three structures of Teun A. van Dijk's discourse analysis model, namely: macrostructure, superstructure, and microstructure.

**Results: **From 1910-1925 there were 24 indigenous newspapers published in North Sumatra. Of these, there were 13 newspapers demanding Indonesian independence: *Soeara Djawa, Pewarta Deli, Benih Merdeka, Perempoean Bergerak, Soeara Bondjol, Sinar Zaman, Orgaan Bataksche Studiefonds, Andalas, Mandailing, Warta Timur, Al Moektabas, Tjermin Karo, Soeara Batak*. These newspapers published articles that fought for Indonesian independence by demanding Indonesian independence openly, criticizing various policies of the Dutch Colonial Government, and building awareness of Indonesian nationalism.

**Conclusion: **The role of newspapers in the struggle for Indonesian independence in North Sumatra in 1916-1925 can be seen from the findings of 51 articles demanding Indonesia's independence from Dutch colonialism, criticizing the policies of the Dutch colonial government, and building the spirit of nationalism to encourage the Indonesian people to fight for their independence.

## Introduction

Newspapers are the oldest form of mass media and have become an important part of modern society which requires the rapid spread of information. ^1^ In the history of Indonesia, newspapers have not only functioned as a medium of information but also as a tool in the struggle for independence. This is tied to the role of newspapers in promoting the spirit of nationalism and the concept of “nation” among the Indonesian people through various writings and reports in each edition. According to Benedict Anderson, ^2^ a nation is a political community that is imagined as something that is both inherently limited and sovereign. The nation did not form by itself there were efforts from the community to create the concept of a nation. Newspapers have an important role in fostering a sense of nationalism and instilling the concept of “nation” for every member of the community.

Nationalism played an important role in the emergence of Indonesia as a country free from colonialism. Anthony D. Smith ^3^ stated that nationalism is an ideological movement that aims to maintain the autonomy, unity, and identity of a nation. It takes a strong determination from its members to make this happen. Hans Kohn ^4^ argues that nationalism grows and develops because of a common desire to unite based on a common destiny, language, and historical journey. Active and real involvement of the people as citizens of equal status, united by a sense of brotherhood (feeling as one family), and loyalty to one another are important moral principles in a nation. This sense of brotherhood is a manifestation of the collective will that has emerged in European nationalism in the eighteenth century. ^3^

The revival of Indonesian nationalism began with access to modern education for indigenous people at the end of the nineteenth century by the Dutch Colonial Government which aimed to produce low-ranking indigenous employees. In 1901 the policy of Ethical Politics began to be implemented in Indonesia, especially to the Javanese people as a reward for their suffering due to a very cruel forced cultivation system. The establishment of the development of education for indigenous people as one of the core programs of *Ethische Politiek* encourages enormous progress and wider opportunities to enter modern European education. *Hollandsch Inlansche School* (HIS), *Meer Uitgebreid Lagger Onderwijs* (MULO), *School Tot Opleiding van Inlandsche Artsen* (STOVIA), dan *Opleiding school voor Inlandsche Ambtenaaren* (OSVIA) are some examples of schools built by the Dutch colonial government for indigenous Indonesian children after the declaration of *Ethische Politiek.* In addition to government schools, there are also private schools owned by Taman Siswa, Muhammadiyah, INS Kayutanam, and Persis starting in the 1920s. The efforts to advance education carried out by the government and also the Indonesian people made education appear as the most important element in the process of progressing Indonesian society at that time. ^5^

The educated group was the core influence in the rise of nationalism in the colonies. ^2^ Modern education introduced by the Dutch colonial government in Indonesia was not only successful in creating educated indigenous people to be employed as part of the colonial bureaucracy but also succeeded in encouraging the birth of pioneer figures for the Indonesian nationalism movement. This phenomenon occurred because the modern European education system at that time gave Indonesian educated people the opportunity to study various nationalist movements in Europe. The European nationalism movement then made these educated people aware that Indonesia was a colonized nation. The suffering of the people due to Dutch colonialism finally encouraged them to fight for Indonesian independence.

The progress of modern education and political movements carried out by educated Indonesians in the early twentieth century correlated with the progress of the Indonesian print press. Newspapers that were introduced by the Dutch colonial government in 1659 for the benefit of their group had an influence on Indonesian educated people at that time. Indigenous intellectual groups eventually created their newspapers. They wrote about nationalist ideas, the importance of unity as a nation, and also the right to independence for the Indonesian people. These educated people also used newspapers as a medium of communication between fellow movement figures to unify the ideas of nationalism and independence that they are fighting for. The modern education system, political organization, and the press media were the three main elements in the formation and dissemination of nationalist ideas in the colonized countries.

The role of newspapers in fostering a sense of nationalism and disseminating the notion of nationalism through newspapers also occurred in North Sumatra, which became one of the centers of newspaper publishing in Indonesia in the early twentieth century. This is evidenced by the number of newspapers published in this region from 1900-1942, reaching more than 140 newspaper titles. ^6^ All of these newspapers had news content and themes that represented various groups. Print media was the most influential in promoting the concept of nationalism. ^2^ The increasing political activity in the national movement among indigenous peoples in North Sumatra from 1916 had a direct impact on newspaper publishing in this area. From 1916 to 1925, 13 newspapers were published to spread the spirit of nationalism and demand Indonesian independence from Dutch colonialism. This study aims to reveal how the forms of Indonesia's struggle for independence were presented in these 13 newspapers.

## Methods

This research is qualitative and uses the critical discourse analysis method. Critical discourse analysis aims to study and analyze the way abuse of social power, dominance, and inequality are enacted, reproduced, and resisted by written and spoken communication in social, political, and historical contexts. ^7^ Critical discourse analysis also aims to find the relationship between causality and determination contained in practices, events, and texts. Power and hegemony are the causative factors of the unclear relationship between these practices, events, and texts. ^8^ Texts play an important role in making history, where texts are evidence of how the historical process takes place. The redefinition of social relations between the professional and the public, the reconstruction of social identities and forms of self, or the reconstruction of knowledge and ideology are evidence of processes that can be observed from texts. ^8^ The texts that are focused on in this research are articles in indigenous newspapers published in North Sumatra in 1916-1925.

The discourse analysis method is appropriate to examine textual data. Teun A. van Dijk divides critical discourse analysis into three structures: macrostructure, superstructure, and microstructure. The macrostructure emphasizes the global meaning of a text that can be observed from the topic or theme appointed by a text. Superstructure focuses on the complete framework of a text which consists of an introduction, content, finale, and conclusion which are systematically arranged. The microstructure is divided into semantics (the meaning of words and sentences that are emphasized in the text), syntax (the form and arrangement of sentences conveyed), stylistics (the choice of words used), and rhetoric (how expressions or language styles are used). ^7^

The data used in this study were texts from 13 indigenous newspapers published in North Sumatra in 1916-1925. All newspaper texts used as data in this research are collections from the National Library of the Republic of Indonesia, the North Sumatra Press Museum, the Medan History House, and the Center for the Study of History and Social Sciences, Universitas Negeri Medan. The texts contained in these newspapers will be analyzed using the three analytical structures of Van Dijk's model described above to find out how the forms of Indonesian independence were portrayed in thirteen newspapers published in North Sumatra in 1916-1925.

## Results

### Publishing Indigenous newspapers in North Sumatra

European influence became the main factor that pushed the press publishing business in North Sumatra forward in the early twentieth century. In 1902 the Dutch began to publish a newspaper for the indigenous people of East Sumatra which was named *Pertja Timor.* In addition to using the Malay language in each edition, the *Pertja Timor* newspaper also provided an opportunity for the indigenous people of North Sumatra to be involved in this newspaper by appointing Mangaradja Salembuwe from Angkola (South Tapanuli) as editor-in-chief. The opportunity to be actively involved in the publishing business for the indigenous people finally prompted the publication of the *Pewarta Deli* newspaper in Medan in 1910. *Pewarta Deli* was the first indigenous newspaper published in North Sumatra, because this newspaper was fully managed by the Indonesians themselves, starting from the shareholders, editors, directors, clerks, and printing presses, all of which belonged to the indigenous people without European intervention. That's why *Perwata Deli* was given the title as the first national newspaper in North Sumatra. ^6^

The emergence of *Pewarta Deli* in the press in North Sumatra eventually prompted the publication of various other indigenous newspapers. Indigenous people have been the most active and most widely published in newspaper publishing in North Sumatra since the publication of *Pewarta Deli* in 1910. Indigenous newspaper publishing in North Sumatra is spread across various regions, from the east coast of Sumatra to the west coast of Sumatra. In Table 1 it can be seen that Medan, which became the capital of the East Sumatra Residency, became an area with as many as twelve names of newspapers. Sibolga, the capital of the Tapanuli Residency, which is located on the west coast of Sumatra, is the second area that publishes the most indigenous newspapers with a total of five newspapers. Seven other indigenous newspapers, published in different areas: *Sinar Merdeka* published in Padang Sidempuan, *Soara Batak* published in Tarutung, *Bataksche Studiefonds* published in Kota Nopan, *Seroean Kita* published in Perbaungan, *Almoektabas* published in Tanjung Balai, *Nias Berita* published in Gunung Sitoli, *Soeara Kita* is published in Pematang Siantar.

**Table 1.** The indigenous newspapers published in North Sumatra (1910-1925). ^6^

No.	Newspaper name	Publication year	Place of publication
1.	*Pewarta Deli*	1910	Medan
2.	*Benih Merdeka*	1916	Medan
3.	*Soeara Djawa*	1916	Medan
4.	*Tapian Na Oeli*	1919	Sibolga
5.	*Soeara Bondjol*	1918	Medan
6.	*Sinar Merdeka*	1919	Padang Sidempuan
7.	*Soara Batak*	1919	Tarutung
8.	*Sama Rata*	1919	Medan
9.	*Perempoean Bergerak*	1919	Medan
10.	*Hindia Sepakat*	1920	Sibolga
11.	*Sinar Zaman*	1921	Medan
12.	*Bataksche Studiefonds*	1922	Kota Nopan
13.	*Pantjaran Berita*	1922	Medan
14.	*Seroean Kita*	1922	Perbaungan
15.	*Warta Timoer*	1923	Medan
16.	*Benih Timoer*	1924	Medan
17.	*Boemipoetera*	1924	Medan
18.	*Almoektabas*	1924	Tanjung Balai
19.	*Pertjatoeran*	1924	Sibolga
20.	*Nias Berita*	1924	Gunung Sitoli
21.	*Soeara Kita*	1925	Pematang Siantar
22.	*Soeara Tapanoeli*	1925	Sibolga
23.	*Oetoesan Sumatra*	1925	Medan
24.	*Poesaka*	1925	Sibolga

The year of publication of the 24 indigenous newspapers in North Sumatra in Table 1 also varies. *Pewarta Deli* became the first indigenous newspaper published in North Sumatra. In 1916, two new indigenous newspapers emerged, namely *Benih Merdeka* and *Soeara Djawa.* In 1918 the *Soeara Bondjol* newspaper appeared. In 1919 five newspapers appeared: *Tapian Na Oeli*, *Sinar Merdeka*, *Soara Batak*, *Sama Rata*, *Perempoean Bergerak.* In 1920 the *Hindia Sepakat* newspaper was published. In 1921 the *Sinar Zaman* newspaper was published. In 1922 three newspapers were published: *Bataksche Studiefonds*, *Pantjaran Berita*, *Seroean Kita.* In 1923 *Warta Timoer* was published. In 1924 the newspapers *Benih Timoer*, *Boemipoetera*, *Almoektabas*, *Pertjatoeran*, *Nias Berita* were published. In 1925 four newspapers were published: *Soeara Kita*, *Soeara Tapanoeli*, *Oetoesan Sumatra*, *Poesaka.*

### Indigenous newspapers as a tool within the Indonesian independence struggle in North Sumatra in 1916-1925

The focus of this research is to find newspapers that feature news about the struggle for Indonesian independence. The first step of data analysis is to read one by one the articles from 24 indigenous newspapers in North Sumatra in Table 1. This first stage uses macrostructural analysis to find topics or themes contained in a text. ^7^ The topic of Indonesia as the colonized and the Dutch as the colonizing became the main guide. The results of the analysis of Table 1 found 13 newspapers that featured news about Indonesia as a Dutch colony. These thirteen newspapers are *Soeara Djawa*, *Pewarta Deli*, *Benih Merdeka*, *Perempoean Bergerak*, *Soeara Bondjol*, *Sinar Zaman*, *Orgaan Bataksche Studiefonds*, *Andalas*, *Mandailing*, *Warta Timur*, *Al Moektabas*, *Tjermin Karo*, *Soeara Batak.* In the thirteen newspapers found as many as 162 articles consisting of various titles and publishing times which can be observed in Table 2. However, the 162 articles have the same theme, namely about Indonesia as a Dutch colony. In terms of the time of publication, Table 2 provides information that the article with the earliest published year was June 1, 1916 ( *Soeara Djawa*) and the article with the latest date was December 24, 1925 ( *Soeara Batak*).

**Table 2.** Indigenous newspapers in North Sumatra (1916-1925) that featuring news about Indonesia as a Dutch colony.

No	Newspaper name	Date of issue	Number of articles
1.	*Soeara Djawa*	6/1/1916 – 7/1/1918	16
2.	*Pewarta Deli*	7/2/1917 – 8/1/1917	7
3.	*Benih Merdeka*	7/2/1918 – 4/8/1920	16
4.	*Perempoean Bergerak*	5/15/1919 – 12/15/1920	22
5.	*Soeara Bondjol*	6/1/1920 – 8/1/1922	4
6.	*Sinar Zaman*	10/8/1921	1
7.	*Orgaan Bataksche Studiefonds*	2/1/1921 – 3/31/1922	3
8.	*Andalas*	11/1/1923 – 11/6/1923	6
9.	*Mandailing*	12/13/1923 – 3/3/1923	15
10.	*Warta Timur*	10/8/1923 – 11/29/1923	16
11.	*Al Moektabas*	1/31/1924	7
12.	*Tjermin Karo*	1/13/1925 – 12/28/1924	8
13.	*Soeara Batak*	2/21/1920 – 12/24/1925	41
Total			162

In Table 2, *Soeara Batak* became the newspaper with the most articles, which amounted to 41 articles published from February 21, 1920, to December 24, 1925. *Perempoean Bergerak* became the second-largest newspaper with 22 articles published from May 15, 19191, to December 15, 1920. *Soeara Djawa*, *Benih Merdeka*, *Warta Timur* took the third position where the articles found in these three newspapers each had 16 article titles. For the time of publication, these three newspapers are different. The article on *Soeara Djaw*a is dated June 1, 1916, to July 1, 1918. *Benih Merdeka* from July 2, 1918, to April 8, 1920. Articles in *Warta Timur* had the shortest period time of one month, from 8 October 1923 to 29 November 1923. The *Mandailing* newspaper had 15 articles published from March 3, 1923, to December 13, 1923. *Tjermin Karo* had eight articles published from December 28, 1924, to January 13, 1925. *Pewarta Deli* and *Al Moektabas* reporters each found seven articles. *Pewarta Deli* was published from July 2, 1917, to August 1, 1917. The seven articles in the *Al Moektabas* were published on the same day, January 31, 1924. *Andalas* newspaper has six article titles with publication dates from November 1, 1923, to November 6, 1923. *Soeara Bondjol* has four articles published from June 1, 1920, to August 1, 1922. *Orgaan Bataksche Studiefonds* had three articles published from February 1, 1921, to March 31, 1922. The last one is that *Sinar Zaman* has only 1 article dated October 8, 1921. Complete information regarding the publication date of each article can be seen in Table 3.

**Table 3.** The result of the analysis of macrostructure, superstructure, and microstructure from indigenous newspapers in North Sumatra in 1916-1925.

No	Newspaper name	Article title	Date of issue	Number of articles
1.	*Soeara Djawa*	The Pen Turns to the Honorable Brother	6/1/1916	3
		The Grand Meeting of Syarikat Islam in Medan Deli	4/1/1918	
		The Movement	6/1/1918	
2.	*Pewarta Deli*	Indies Nederland	7/2/1917	3
		The welfare of the North Sumatran people can be lost	7/27/1917	
		Newspaper	7/27/1917	
3.	*Benih Merdeka*	Liberate the nation	5/29/1918	6
		Memory Fate!	5/30/1918	
		Reckless	7/2/1918	
		Soekidjo	8/29/1918	
		General Court	3/25/1920	
		Land for the People	3/25/1920	
4.	*Perempoean Bergerak*	Deliberation	7/16/1919	4
		Marriage to another Nation	12/16/1919	
		The Cry of the Little One	2/15/1920	
		Our Feelings	11/15/1920	
5.	*Soeara Bondjol*	Love the homeland	6/1/1920	1
6.	*Sinar Zaman*	Brain, Blood, and Unity of the Heart	10/8/1921	1
7.	*Orgaan Bataksche Studiefonds*	The position of Mr. Dr. Abdul Rasjid (To demand the progress of the country's children in the Tapanuli Residency to follow the right path)	2/1/1921	1
8.	*Andalas*	Mandjono is too brave	11/3/1923	1
9.	*Mandailing*	The aspired progress	1/27/1923	10
		Forced Labor Money and the Situation of the Village	2/17/1923	
		The World Movement of the Mandailing Nation	2/17/1923	
		British Indies	1/13/1923	
		Intelligence and independence	1/27/1923	
		Independence in Movement	2/3/1923	
		Pan Islamism	2/3/1923	
		When will the world be safe?	2/17/1923	
		Aceh blood	2/24/1923	
		Yours faithfully	3/3/1923	
10.	*Warta Timur*	The emergence of a dark case in the political workgroup in Betawi	10/18/1923	3
		It's getting harder!	11/26/1923	
		Help! Help! Help!	11/29/1923	
11.	*Al Moektabas*	Aspirations and Feelings	1/31/1924	3
		Homeland and nation	1/31/1924	
		The Principles of Nation's Progress	1/31/1924	
12.	*Tjermin Karo*	The Call (especially for my Batak Karo brothers)	11/13/1924	3
		A brief statement from Langkat Hulu	12/23/1924	
		Hope	1/13/1925	
13.	*Soeara Batak*	Economic movements in Tapanuli	3/6/1920	12
		Distant sound	3/13/1920	
		Love the Nation	3/13/1920	
		Four questions and one suggestion	3/12/1921	
		The Matter of Land and the IR Nation (Ireland and Irishmen)	8/20/1921	
		Achieving Independence	8/27/1921	
		The Great Meeting	1/21/1922	
		Is it true that Indonesia will soon be given independence by the Dutch	1/27/1923	
		The Ideals of Independence	4/7/1923	
		Desire to be noticed by the government	7/13/1925	
		What causes our association to become weak?	8/22/1925	
		Flying from Europe to America	11/21/1925	
Total				51

The next stage is to analyze the 162 articles in Table 2 using macrostructure, superstructure, and microstructure analysis ^7^ in stages to find which articles contain news about the struggle for Indonesian independence. The results of the analysis found that there were only 51 articles featuring news about the Indonesian independence struggle which can be observed in Table 3.

In Table 3 *Soeara Batak* became the newspaper with the most article titles, namely 12 articles: three articles published in 1920, three articles in 1921, one article published in 1922, two articles published in 1923, and three articles published in 1925. The *Mandailing* newspaper had 10 articles published in the same year with details: one article published on January 13, 1923, two articles published on January 27, 1923, two articles published on February 3, 1923, three articles published on February 17, 1923, one article published on February 24, 1923, and one article appeared on March 3, 1923. *Benih Merdeka* has six article titles: four articles published in 1918 and two articles published in 1920 on the same publication date. The *Perempoean Bergerak* newspaper has four articles with details: two articles published in 1919 and two articles published in 1920. In *Soeara Djawa* there are three titles of articles published in different editions, namely one article in 1916 and two articles in 1918. *Pewarta Deli* also has three articles published in the same year, namely 1917. Two articles published by *Pewarta Deli* were published on the same publication date, namely 27 July 1917. *Warta Timur* also has three articles published in 1923, one in October and two in November. *Al Moektabas* also has three titles of articles published in the same edition on January 31, 1924. The *Tjermin Karo* newspaper had two articles published in 1924 and one article published in 1925. *Soeara Bondjol*, *Sinar Zaman*, *Orgaan Bataksche Studiefonds*, and *Andalas* each had only 1 article title published in 1920, 1921, and 1923.

The macrostructure is the first stage of analysis carried out to get the results from Table 3. Macrostructure only analyzes the surface or general things of a text. The topic or theme of the text is used as an indicator in this data analysis process. The struggle for Indonesian independence is the topic used. This analysis produces data from articles and newspapers that display news about Indonesian independence in ‘the macrostructural analysis results from indigenous newspapers in North Sumatra (1916-1925)’ (see underlying data: 10.6084/m9.figshare.17894687).

The macrostructural analysis results in ‘the macrostructural analysis results from indigenous newspapers in North Sumatra (1916-1925)’ (see underlying data: 10.6084/m9.figshare.17894687) show that the titles of the articles in the indigenous newspapers in North Sumatra published in 1916-1925 did not openly write about the demands for Indonesian independence. However, when the topic of the text is found, the statement regarding the demands or struggle for Indonesian independence is very clear. This can be observed in the *Soeara Djawa* newspaper with the article title “The Pen Turns to the Honorable Brother” which turns out to have a topic to support the era of movement. “The era of movement” refers to the period of the struggle for independence that took place from 1900-1942. In the article “Memory Fate!” in the *Benih Merdeka* newspaper, the topic was Indonesia can be independent. *Al Moektabas* has an article entitled “Aspirations and Feelings” with the topic tax rules in the city of Tanjung Balai are a form of protest against the high taxes that people have to pay to the Dutch colonial government. If examined one by one from the titles and topics written in Table 4, there are hidden or implicit messages that the author wants to convey regarding the struggle for Indonesian independence.

The next stage is to conduct a superstructure analysis (second stage analysis) using the data in ‘the macrostructural analysis results from indigenous newspapers in North Sumatra (1916-1925)’ (see underlying data: 10.6084/m9.figshare.17894687). Superstructure analysis analyzes the text framework which consists of the introduction, content, and closing. The three parts of the superstructure contain core information or specific data that a text ^9^ wants to convey in the form of "premises" and “conclusions”. ^10^ Therefore, at this stage a more in-depth analysis is carried out on what messages or information are in the introduction, content, and closing sections of the 51 articles in Table 3. The discourse on the struggle for Indonesian independence becomes the focus of this superstructure analysis.

The results of the analysis succeeded in finding out which part of each article contained a key statement, namely the struggle for Indonesian independence. ‘The superstructure analysis results from indigenous newspapers in North Sumatra (1916-1925)’ (see underlying data: 10.6084/m9.figshare.17894687) that the most frequently found key statements are at the beginning of the text, which amounts to 30 citations. Next is at the end of the text with 16 citations. The number of key statements found at least in the middle of the text is 10 citations.

In *Benih Merdeka* with the article title “Memory Fate”, key statements are found at the beginning, middle, and end of the text. The article entitled “Land for the People” published in the newspaper *Benih Merdeka* has key statements only at the beginning and end of the text. The article entitled “Aspirations and Feelings” has key statements at the beginning and middle of the text. In the article entitled “Hope” the key statements are found at the beginning and end of the text. ‘The superstructure analysis results from indigenous newspapers in North Sumatra (1916-1925)’ (see underlying data: 10.6084/m9.figshare.17894687) also shows that the article “We are responsible! We demand!” found two key statements at the end of the text. Meanwhile in the article entitled “The Matter of Land and the IR Nation (Ireland and Irishmen)” two key statements were found at the beginning of the text. Other articles contain only one key statement at one point in the text.

After the superstructure stage has succeeded in finding the location where the key statement is in a text, the third step is to conduct a microstructural analysis to identify whether the text discusses the struggle for Indonesian independence. The results of the microstructural analysis are the use of words, phrases, or sentences from each article. These words, phrases, or sentences are the essences of the discourse the writer wants to convey to the readers. Readers must understand every word, phrase, and sentence that is often conveyed implicitly by the author in every article written. As a result, the title of the article and the results of the analysis of the microstructure in the column “Use of words/phrases” do not seem to have anything in common with each other.

The results of the microstructural data analysis in ‘The microstructural analysis results from indigenous newspapers in North Sumatra (1916-1925)’ (see underlying data: 10.6084/m9.figshare.17894687) show that there are articles that contain unequal amounts of key statements in the form of words, phrases, sentences, or a combination of the three. Most of the key statements were found in the *Perempoean Bergerak* newspaper with the article title “Marriage to another Nation” with six quotes. The articles entitled “Soekidjo” and “Land for the People” both contain four quotes.

Thirteen article titles in ‘The microstructural analysis results from indigenous newspapers in North Sumatra (1916-1925)’ (see underlying data: 10.6084/m9.figshare.17894687) each have three citations: The Grand Meeting of Syarikat Islam in Medan Deli, Newspaper, Reckless, Our Feelings, Brain, Blood, and Unity of the Heart, The position of Mr. Dr. Abdul Rasjid (To demand the progress of the country's children in the Tapanuli Residency to follow the right path), Forced Labor Money and the Situation of the Village, The World Movement of the Mandailing Nation, The Call (especially for my Batak Karo brothers), Economic movements in Tapanuli, Distant sound, The Matter of Land and the IR Nation (Ireland and Irishmen), dan The Great Meeting. Meanwhile, the article which has two citations include: The Pen Turns to the Honorable Brother, Indies Nederland, We are responsible! We demand! Memory Fate! Deliberation, Mandjono is too brave, The aspired progress, British Indies, Intelligence and independence, Independence in Movement, Aceh blood, Yours faithfully, The emergence of a dark case in the political workgroup in Betawi, It's getting harder! Homeland and nation, The Principles of Nation's Progress, Hope, Love the Nation, Four questions and one suggestion, Achieving Independence, The Ideals of Independence, Flying from Europe to America.

Articles containing only one citation in ‘The microstructural analysis results from indigenous newspapers in North Sumatra (1916-1925)’ (see underlying data: 10.6084/m9.figshare.17894687) are entitled: the movement, the welfare of the North Sumatra people can be lost, general court, the cry of the little one, love the homeland, pan Islamism, when will the world be safe? Help! Help! Help! Aspirations and feelings, a brief statement from Langkat Hulu, is it true that Indonesia will soon be given independence by the Dutch, desire to be noticed by the government, and what causes our association to become weak?

The microstructural analysis also succeeded in categorizing 51 articles in Table 3 into three groups, namely 1) demanding Indonesian independence openly, 2) criticizing various policies of the Dutch Colonial Government to the indigenous people of North Sumatra, 3) building awareness of nationalism through the dissemination of the spirit of nationalism, unity, and the Indonesian independence movement.

1. Demanding Indonesian independence openly.

There are twelve articles in four newspapers openly wrote about the demands for Indonesian independence. Details of the 12 newspapers whose contents openly demand Indonesian independence can be seen in Table 4.

**Table 4.** The newspapers that demand Indonesian independence openly.

No	Newspaper name	Date of issue	Article title
1.	*Benih Merdeka*	5/30/1918	Memory Fate!
2.	*Benih Merdeka*	3/16/1920	We are responsible! We demand!
3.	*Soeara Djawa*	6/1/1918	The Movement
4.	*Mandailing*	1/13/1923	British Indies
5.	*Mandailing*	1/27/1923	Intelligence and independence
6.	*Mandailing*	2/3/1923	Independence in Movement
7.	*Mandailing*	2/3/1923	Pan Islamism
8.	*Mandailing*	2/17/1923	When will the world be safe?
9.	*Soeara Batak*	8/27/1921	Achieving Independence
10.	*Soeara Batak*	1/21/1922	The Great Meeting
11.	*Soeara Batak*	1/27/1923	Is it true that Indonesia will soon be given independence by the Dutch?
12.	*Soeara Batak*	4/7/1923	The Ideals of Independence

In Table 4 it can be observed that the *Mandailing* newspaper has the most articles demanding Indonesian independence, namely as many as five articles with the title “British Indies”, “Intelligence and independence”, “Independence in Movement”, “Pan Islamism”, dan “When will the world be safe?”. The next most articles were found in *Soeara Batak* as many as four articles with the title “Achieving Independence”, “The Great Meeting”, “Is it true that Indonesia will soon be given independence by the Dutch?” dan “The Ideals of Independence”. *Benih Merdeka* has two articles with the title “Memory Fate!” dan “We are responsible! We demand!” In the *Soeara Djawa* newspaper, only one article was found entitled “The Movement”.

For the year of publication, Table 4 can be seen that 1923 was the most widely published article demanding Indonesian independence, of which there are as many as seven articles. 1923 was also the last year to publish an article on this theme. The earliest year is 1918 with two article titles. The years 1920, 1921, 1922 each had one article.

The results of the twelve newspapers which are included in the category of demanding Indonesian independence openly are based on the results of the microstructural analysis in the form of the use of words, phrases, and sentences in the text. For more details regarding the results of the microstructural analysis of the twelve articles in Table 4, it can be seen in Table 5 below.

**Table 5.** The microstructural analysis results from the newspapers that openly demand Indonesian independence.

Article title	The quotation of the text
Memory Fate!	- The Indies nation (Indonesian) can be independent - The land of the Indies wants to be independent
We are responsible! We demand!	An alliance that wants to establish an indigenous (Indonesian) government
The Movement	To advance the economy and independence of the Indies
British Indies	Demanding the independence of the nation and establishing the Republic of Indonesia
Intelligence and independence	The people of the Indies (Indonesia) ask for their independence
Independence in Movement	Independence will move to achieve independence
Pan Islamism	How did the land of the Indies (Indonesia) become independent?
When will the world be safe?	Hundreds of Indonesians imprisoned for demanding independence
Achieving Independence	Work hard for the progress and independence of the nation
The Great Meeting	- The desire for national independence - Not subject to any power
Is it true that Indonesia will soon be given independence by the Dutch	Indonesia will not be a nation that will be colonized forever
The Ideals of Independence	Free from Dutch colonial rule

In Table 5 the demands for Indonesian independence in the newspaper *Benih Merdeka* through an article with the title “Memory Fate!” is seen in the use of the sentences “The Indies nation (Indonesian) can be independent” and “The land of the Indies wants to be independent”. Next up in the article entitled “We are responsible! We demand!”, the author wrote the sentence “An alliance that wants to establish an indigenous (Indonesian) government”. The article entitled “The Movement” demands Indonesian independence through the sentence “To advance the economy and independence of the Indies”. The article entitled “British Indies” wrote, “Demanding the independence of the nation and establishing the Republic of Indonesia”. If we examine one by one Table 8 in the quotation of the text column, it is seen that the choice of words, phrases, or sentences used openly and firmly in the article demands Indonesia's independence from Dutch colonialism.

2. Criticizing various policies of the Dutch Colonial Government to the indigenous people of North Sumatra.

Writings containing criticism and protests against the Dutch colonial government became the next category found from the microstructural analysis of 51 articles in Table 3. The results of the analysis found 19 article titles whose contents were about criticism and protest against all the policies of the Dutch colonial government which were considered to be oppressing the indigenous people. In addition, there are suggestions and requests for the colonial government to do things that can advance the indigenous people. Details of the 19 titles of articles that criticize the Dutch colonial government can be seen in Table 6 below.

**Table 6.** The newspapers that criticized the colonial government's policies.

No	Newspaper name	Date of issue	Article title
1.	*Soeara Djawa*	4/1/1918	The Grand Meeting of Syarikat Islam in Medan Deli
2.	*Pewarta Deli*	7/2/1917	Indies Nederland
3.	*Benih Merdeka*	7/2/1918	Reckless
4.	*Benih Merdeka*	8/29/1918	Soekidjo
5.	*Benih Merdeka*	3/25/1920	General Court
6.	*Benih Merdeka*	3/25/1920	Land for the People
7.	*Perempoean Bergerak*	7/16/1919	Deliberation
8.	*Perempoean Bergerak*	12/16/1919	Marriage to another Nation
9.	*Perempoean Bergerak*	2/15/1920	The Cry of the Little One
10.	*Perempoean Bergerak*	11/15/1920	Our Feelings
11.	*Warta Timur*	10/18/1923	The emergence of a dark case in the political workgroup in Betawi
12.	*Warta Timur*	11/26/1923	It's getting harder!
13.	*Warta Timur*	11/29/1923	Help! Help! Help!
14.	*Mandailing*	2/17/1923	Forced Labor Money and the Situation of the Village
15.	*Al Moektabas*	1/31/1924	Aspirations and Feelings
16.	*Tjermin Karo*	1/13/1925	Hope
17.	*Soeara Batak*	3/12/1921	Four questions and one suggestion
18.	*Soeara Batak*	8/20/1921	The Matter of Land and the IR Nation (Ireland and Irishmen)
19.	*Soeara Batak*	7/13/1925	Desire to be noticed by the government

In Table 6 above, there are nine indigenous newspapers in North Sumatra which contain articles criticizing the Dutch colonial government. The newspapers of *Benih Merdeka* and *Perempoean Bergerak* became the newspapers with the highest number of articles, each with four articles. The next most articles with three article titles are *Warta Timur* and *Soeara Batak.* The newspapers of *Soeara Djawa*, *Pewarta Deli*, *Mandailing*, *Al Moektabas*, *Tjermin Karo* only had one article title.

The year of publication of the article with the theme of criticism of the Dutch colonial government in Table 6 began in 1917 with an article entitled “Indies Nederland”. In 1918 there were three article titles. In 1919 two article titles. In 1920 there were as many as four article titles. In 1921 there were two article titles. In 1923 there were four article titles. In 1924 there was one article title. In 1925 there were as many as two article titles. The results of the microstructural analysis of the 19 articles in Table 6 can be observed in Table 7.

**Table 7.** The microstructural analysis results from the newspapers that criticizing the colonial government's policies.

Article title	The quotation of the text
The Grand Meeting of Syarikat Islam in Medan Deli	People cannot speak in court and are immediately punished
Indies Nederland	Arbitrary acts of Dutch officials
Reckless	The government does not care about the interests of the people
Soekidjo	Rulers oppress people so much that people's lives are very miserable
General Court	The rule of forced labor ( *rodi*) must be immediately abolished by the Dutch colonial government
Land for the People	The Dutch colonial government was unable to protect the people from the growing misery
Deliberation	The Dutch colonial government regulations do not need to be followed because they are contrary to the customs and religion of the people of North Sumatra
Marriage to another Nation	The opening of foreign plantations carried out by the Dutch colonial government had damaged the security of the people, seized the people's independence, robbed the people of pleasure, removed the people's glory, was hostile to the people's religion, and destroyed the walls of national boundaries.
The Cry of the Little One	The Dutch colonial government should have raised the salaries of indigenous employees appropriately
Our Feelings	The obligation to pay taxes to the Dutch colonial government brought misery to the Indonesia people
The emergence of a dark case in the political workgroup in Betawi	Convey information to the government that the poor are getting hungry and they are still required to pay taxes
It's getting harder!	The life of the Indonesia people is not getting more advanced, but getting more difficult
Help! Help! Help!	Government! Look and pay attention to your people who ask for help
Forced Labor Money and the Situation of the Village	What was the forced labor tax money used by the Dutch colonial government?
Aspirations and Feelings	The colonial government's tax rules made people's lives more difficult
Hope	- It's time to estimate the tax rate for 1925 - The rulers (Dutch colonial government)! Have mercy on your people who are in trouble
Four questions and one suggestion	Hopefully, the government will release M.H. Manullang
The Matter of Land and the IR Nation (Ireland and Irishmen)	Compare the situation between the businessmen who were supported by the Dutch colonial government and the poor and ignorant native farmers
Desire to be noticed by the government	The longer the poorer the life of the Bataklanden people

Table 7 shows that the criticism of the Dutch colonial government in the *Soeara Djawa* newspaper with the article title “The Grand Meeting of Syarikat Islam in Medan Deli” can be seen in the use of the sentence “People cannot speak in court and are immediately punished”. In the *Pewarta Deli* newspaper entitled “Indies Nederland” the sentence “Arbitrary acts of Dutch officials” was written. In *Warta Timur* newspaper entitled “Help! Help! Help!” the author submits a request to the Dutch colonial government through the sentence “Government! Look and pay attention to your people who ask for help”. The contents of the news regarding suggestions and requests to the Dutch colonial government are also contained in the article entitled “The Cry of the Little One” which writes “The Dutch colonial government should have raised the salaries of indigenous employees appropriately”.

3. Building awareness of nationalism through the dissemination of the spirit of nationalism, unity, and the Indonesian independence movement.

Building awareness of nationalism through the dissemination of the spirit of nationalism, unity, and the Indonesian independence movement is the last category of indigenous newspapers that fight for Indonesian independence. The results of the microstructural analysis managed to find twenty articles published in ten newspapers. The ten newspapers and twenty article titles can be seen in Table 8.

**Table 8.** The newspapers that spread the spirit of nationalism, unity, and the Indonesian independence movement.

No	Newspaper name	Date of issue	Article title
1.	*Soeara Djawa*	6/1/1916	The Pen Turns to the Honorable Brother
2.	*Pewarta Deli*	7/27/1917	The welfare of the North Sumatran people can be lost
3.	*Pewarta Deli*	7/27/1917	Newspaper
4.	*Soeara Bondjol*	6/1/1920	Love the homeland
5.	*Sinar Zaman*	10/8/1921	Brain, Blood, and Unity of the Heart
6.	*Orgaan Bataksche Studiefonds*	2/1/1921	The position of Mr. Dr. Abdul Rasjid (To demand the progress of the country's children in the Tapanuli Residency to follow the right path)
7.	*Mandailing*	1/27/1923	The aspired progress
8.	*Mandailing*	2/17/1923	The World Movement of the Mandailing Nation
9.	*Mandailing*	2/24/1923	Aceh blood
10.	*Mandailing*	3/3/1923	Yours faithfully
11.	*Andalas*	11/3/1923	Mandjono is too brave
12.	*Al Moektabas*	1/31/1924	Homeland and nation
13.	*Al Moektabas*	1/31/1924	The Principles of Nation's Progress
14.	*Tjermin Karo*	11/13/1924	The Call (especially for my Batak Karo brothers)
15.	*Tjermin Karo*	12/23/1924	A brief statement from Langkat Hulu
16.	*Soeara Batak*	3/6/1920	Economic movements in Tapanuli
17.	*Soeara Batak*	3/13/1920	Distant sound
18.	*Soeara Batak*	8/22/1925	What causes our association to become weak?
19.	*Soeara Batak*	11/21/1925	Flying from Europe to America
20.	*Soeara Batak*	3/13/1920	Love the Nation

Based on Table 8 above, *Soeara Batak* is the newspaper that has the highest number of articles, namely five article titles: “Economic movements in Tapanuli”, “Distant sound”, “What causes our association to become weak?”, “Flying from Europe to America, Love the Nation”. *Mandailing* is the newspaper that has the second most articles with the title: “The aspired progress”, “The World Movement of the Mandailing Nation”, “Aceh blood”, “Yours faithfully”. The *Pewarta Deli* newspaper has two articles with the titles: “The welfare of the North Sumatra people can be lost” dan “Newspaper”. *Al Moektabas* also has two titles, namely: “Homeland and nation” dan “The Principles of Nation's Progress”. *Tjermin Karo* also has two titles: “The Call (especially for my Batak Karo brothers)” and “A brief statement from Langkat Hulu”. The *Soeara Djawa* newspaper entitled “The Pen Turns to the Honorable Brother”. The *Soeara Bondjol* newspaper entitled “Love the homeland”. The *Sinar Zaman* newspaper is entitled “Brain, Blood, and Unity of the Heart”. The *Orgaan Bataksche Studiefonds* newspaper entitled “The position of Mr. Dr. Abdul Rasjid (To demand the progress of the country's children in the Tapanuli Residency to follow the right path)”. The *Andalas* newspaper entitled “Mandjono is too brave”.

Table 8, 1923, and 1920 were the years with the most articles, namely five articles each. Meanwhile, 1924 had four article titles. The years 1917, 1921, and 1925 each had two article titles. The year 1916 was the earliest and at least one article title. The results of the microstructural analysis of the twenty article titles in Table 11 can be observed in Table 9.

**Table 9.** The results of the microstructural analysis from the newspapers that spread the spirit of nationalism, unity, and the Indonesian independence movement.

Article title	The quotation of the text
The Pen Turns to the Honorable Brother	Together we support this new movement
The welfare of the North Sumatran people can be lost	Don't follow Dutch customs
Newspaper	The injustice experienced by the natives feels like humiliation and stupidity
Love the homeland	- Believe and be sincere to their country - Gone all worries and doubts to his country
Brain, Blood, and Unity of the Heart	Stronger to face the obstacles to the efforts of the independence movement
The position of Mr. Dr. Abdul Rasjid (To demand the progress of the country's children in the Tapanuli Residency to follow the right path)	Unity leads to sufficiency, all-sufficiency leads to ability.
The aspired progress	Let's together establish an association for the benefit of the nation
The World Movement of the Mandailing Nation	The age of movement requires everyone to move to defend and advance their nation and homeland
Aceh blood	The hatred of the Acehnese towards the Dutch
Yours faithfully	Men and women must unite for the independence movement
Mandjono is too brave	I am not afraid of Dutch power
Homeland and nation	The way to love our homeland is to love the place where we were born
The Principles of Nation's Progress	The progress of the nation is the progress made together
The Call (especially for my Batak Karo brothers)	Let's unite for common progress
A brief statement from Langkat Hulu	Very sad to remember the fate of our nation who always lives in misery and humiliation
Economic movements in Tapanuli	Show love for the homeland to the nation
Distant sound	Uniting thoughts for the progress of the nation
What causes our association to become weak?	Proof of love for the nation and love for the homeland
Flying from Europe to America	The strong spirit of the soul will encourage the progress of a nation
Love the Nation	So that Indonesian youth love their nation and homeland

In Table 9 it can be observed that efforts to build awareness of the nationalism of the people of North Sumatra can be seen from the article entitled “The welfare of the North Sumatran people can be lost”. In this article, the author uses the phrase “Don't follow Dutch customs”. The article entitled “Aceh blood” also aims to build a sense of nationalism for readers through the sentence “The hatred of the Acehnese towards the Dutch”. The message regarding national unity can be seen in the article “The Principles of the Nation's Progress” through the sentence “The progress of the nation is the progress made together”. The article entitled “The Call (especially for my Batak Karo brothers)” is also about national unity who written: “Let's unite for common progress”. In the article entitled “Distant sound it is written: Uniting thoughts for the progress of the nation”. The article entitled “The Pen Turns to the Honorable Brother” wrote the sentence “Together we support this new movement” to support the Indonesian independence movement. The article entitled “Brain, Blood”, and “Unity of the Heart” is also related to the Indonesian independence movement through the sentence “Stronger to face the obstacles to the efforts of the independence movement.”

## Discussion

This study aims to describe how the forms that the Indonesian struggled for independence took in 13 indigenous newspapers published in North Sumatra between 1916-1925. Based on the results of the study, of the 162 titles of articles (texts) that were collected during the research, 51 titles of articles were produced whose news content was fighting for Indonesian independence. The 51 article titles were categorized into three groups: 1) demanding Indonesian independence openly, 2) criticizing various policies of the Dutch Colonial Government, 3) building awareness of Indonesian nationalism.

Twelve articles write openly on the topic of Indonesian independence to demand change. This number is the least compared to the other two categories in the struggle for Indonesian independence. Microstructural analysis based on the meaning of words and sentences emphasized in the text, the form and structure of the sentences presented, the choice of words used, and the expression or style of language explicitly shows the demand for Indonesian independence in twelve articles with this theme. On June 1, 1918, *Soeara Djawa* newspaper reported on the establishment of an organization called “Werklub” which aims to promote the economy and independence of Indonesia. Meanwhile, *Mandailing* newspaper dated January 27, 1923, in the articles “Intelligence and independence”, wrote that many Indonesians wanted Indonesian independence. *Soara Batak* dated April 7, 1923, in the article “The Ideals of Independence” appealed to all the people of the Indies to unite forces to achieve independence from Dutch colonialism.

Nineteen articles were found that criticize the colonial government's policies towards indigenous people in North Sumatra in 1916-1925. Tax rules for the people of North Sumatra became the most discussed topic, namely *Warta Timur* on 18 October 1923 and 29 November 1923, *Mandailing* on 17 February 1923, *Al Moektabas* on 31 January 1924, *Tjermin Karo* on 13 January 1925, and *Soeara Batak* on 20 August 1921. Other articles include the poverty of the people of North Sumatra, the negative impact of plantation capitalization on the people of North Sumatra, as well as the arrest of leaders of North Sumatra who are considered to be against the Dutch colonial government.

Twenty articles spread the spirit of nationalism for the people of North Sumatra. The call for “Unity” for the people of North Sumatra to fight against the Dutch colonial government became the most widely raised topic to spread the spirit of nationalism. *Sinar Zaman* on 8 October 1921, *Orgaan Bataksche Studiefonds* on 1 February 1921, *Mandailing* on 27 January 1923, 17 February 1923, and 3 March 1923, *Soeara Bondjol* on 20 June 1923, *Al Moektabas* on 31 January 1924, *Tjermin Karo* on 13 November 1924 and December 23, 1924, and *Soeara Batak* dated March 6, 1920, were articles that wrote about “Unity” in their coverage. Other writings about nationalism are love for the homeland and encouragement to the people of North Sumatra to support the national progressive movement that was taking place in Indonesia at that time.

In the history of Indonesian independence, the second and third decades (1910-1930) were known as the period of the radical political movement of the Indonesian people. This period was marked by the emergence of organizations and newspapers that openly declared independence from Dutch colonial rule. The opening of opportunities in access to modern education in the early 1900s created a group of educated people who were able to read and write. This intellectual group founded political organizations and published indigenous newspapers in Indonesia. Until 1913, Indonesian intellectuals who became publishers and editors of newspapers had realized the importance of “national unity” as the central message of their publications. ^11^ In North Sumatra, militancy in newspapers in the struggle for Indonesian independence was started by the newspaper *Benih Merdeka*, which was first published on November 20, 1916.

For the Dutch Colonial Government, the courage of the indigenous newspapers in demanding Indonesian independence was considered a threat to the stability of their power over their colonies. Therefore, the Dutch Colonial Government established rules for publishing newspapers published in the *Strafwetboek* (Book of Criminal Law). Article 66a of *Strafwetboek* states that anyone who spreads hostility, hatred, or insults to the Dutch government or the Dutch East Indies, either through writing, pictures, or actions will be punished with imprisonment for 5-10 years. Meanwhile, article 66b of *Strafwetboek* states that anyone who spreads hostility, hatred, or insults among the inhabitants of the Dutch East Indies, either through writing, pictures, or actions will be sentenced to imprisonment for six days to five years. ^5^

The rules for publishing the printed press on *Strafwetboek* did not make the indigenous press figures of North Sumatra afraid to use newspapers as a tool for their struggle. This courage was evidenced by the publication of an article on the theme of nationalism in the newspaper *Soara Djawa* on June 1, 1916 (see Table 6). In this article, the author asks all Indonesians to support the ongoing movement. The movement referred to in this newspaper is progress which is defined as an effort to improve the lives of the Indonesian people in the economic, social, cultural, and political fields so that they are no longer inferior. This progress made the Indonesian people able to liberate themselves from Dutch colonialism. This spirit of progress has prompted many articles on the theme of the spirit of nationalism, unity, and the Indonesian independence movement to be found in this research.

The establishment of various political organizations that carried the spirit of the independence movement contributed to the increasing role of newspapers as a tool for the struggle for independence in North Sumatra. The role of newspapers as voice carriers for political organizations makes this mass media very important in disseminating the ideology of indigenous political organizations to the wider community. Newspapers based on political organizations in North Sumatra are *Benih Merdeka* (published by Sarekat Islam (SI) Medan branch), *Soara Batak* (published by Hatopan Kriten Batak (HKB) in Tarutung), ^12^ *Soeara Djawa* (published by Budi Utomo (BU) Pangkalan Berandan branch). ^5^

The above description shows that the print media is the most important element in shaping the concept of nationalism of a nation. Print media is also the fastest way to spread the idea of nationalism to all its members. ^2^ Therefore, print media is often used as a means to shape and spread the notion of nationalism in colonized countries and also as a tool for the struggle for independence. The role of newspaper publishing in the struggle for independence in North Sumatra from 1916 to 1925 can be seen in the three forms of the struggle for Indonesian independence that appear in the thirteen newspapers that have been described. The people of North Sumatra use newspapers as a tool for the struggle for Indonesian independence to the fullest through 51 articles found in this study.

## Conclusion

North Sumatra became a publishing center for indigenous newspapers during the Dutch colonial period. In 1900-1942 there were hundreds of newspapers published in this area. The most important thing about publishing this newspaper is the role of newspapers as a tool for the struggle for independence against Dutch colonialism. The results of research from thirteen indigenous newspapers published from 1916-1925 found that 51 articles contained writings about the struggle for Indonesian independence. Based on the analysis of Teun A. van Dijk's critical discourse which consists of macrostructure, superstructure, and microstructure used in the texts of the thirteen newspapers, 51 articles fight for Indonesian independence. These 51 articles can be categorized into three groups: 1) demanding Indonesian independence openly, 2) criticizing various policies of the Dutch Colonial Government, 3) building awareness of Indonesian nationalism. People in North Sumatra use newspapers as a tool for the struggle for independence as much as possible. They bravely resisted the colonial government through the writings they published, even though the Dutch colonial government would punish them for their resistance.

## Data availability

### Underlying data

The sources of newspapers in our research exist and can be referred to by other researchers at the Jakarta National Library using their website https://www.perpusnas.go.id/ or contact the email below materjilperpusnas20@gmail.com The librarian on duty will answer the email sent to this email address. In addition, these sources can also be seen (offline access only) at the North Sumatra Press Museum, Sei Alas Road No. 6, Medan. The Medan History House, Kota Cina/Pematang Siombak Road, No. 65, Neighborhood 7, Paya Pasir Village, Medan Marelan District, Medan 20250. The Center for the Study of History and Social Sciences (PUSSIS) Universitas Negeri Medan, Willem Iskandar Road, Pasar V, Medan Estate.

This project also contains data available under the term of figshare (CC BY 4.0):

• The macrostructural analysis results from indigenous newspapers in North Sumatra (1916-1925). 10.6084/m9.figshare.17894687
• The superstructure analysis results from indigenous newspapers in North Sumatra (1916-1925). 10.6084/m9.figshare.17894687
• The microstructural analysis results from indigenous newspapers in North Sumatra (1916-1925). 10.6084/m9.figshare.17894687

### Extended data

Figshare: Table of indigenous newspapers published in North Sumatra in 1916-1925 10.6084/m9.figshare.17894687

## Grant Information

This research was funded by DIPA fund of Universitas Negeri Medan for the 2020 Fiscal Year under the UNIMED Rector's Decree No.116/UN33.8/PL-PNBP/2020 dated June 30, 2020.

## Competing interests

No competing interests were disclosed.
