# The role of newspapers published in North Sumatra during Indonesia's independence struggle between 1916-1925: A content analysis

**DOI:** 10.12688/f1000research.53442.2

**Published:** 2023-10-06

**Authors:** Ichwan Azhari, Ricu Sidiq, Ika Purnamasari

**Affiliations:** 1 Department of Historical Education, Medan state university, Medan, Sumatera Utara, 20225, Indonesia

**Keywords:** Newspapers, nationality, independence, North Sumatra

## Abstract

**Background:** Newspapers played an important role in fostering a sense of nationalism, instilling the concept of "nation", fostering a sense of belonging, and maintaining the unity that the colonized people desperately needed to achieve independence. This study aims to investigate the involvement of indigenous newspapers in the struggle for Indonesian independence in North Sumatra during the Dutch colonial period, as well as to highlight how the contents of the newspaper's coverage were narrated in the struggle for Indonesian independence.

**Methods: **This study used a qualitative content analysis (QCA) method. Research analysis includes manifest analysis (shown on the surface of the text) and latent analysis (hidden in the text) on the title, topic and sentence of each article. The validity test was carried out by peer-reviewing coding books and coding forms. Reliability test in the form of Intercoder reliability using NVivo 12 interrater reliability.
** **

**Results:  **The results of the study found 107 articles in 13 indigenous newspapers in North Sumatra that supported the struggle for Indonesian independence from Dutch colonialism. There are 7 forms of narrative of the struggle for Indonesian independence in indigenous newspapers in North Sumatra from 1916-1925. The seven narratives are found in the title, topic, and sentences written in the article. National movements were the most narratives found with 36.73% found in titles (N= 49), 26.09% in topics (N=89), and 23.72% in sentences in the text (N=156). The fewest narrations were criticisms and demands to the Netherlands of 2.04% for title analysis. The next least narrative is patriotism with a score of 5.43% for topic analysis and 6.41% for sentences in the text.
** **

**Conclusion: **The role of newspapers in the struggle for independence in North Sumatra is evidenced by the findings of 107 articles in 13 newspapers in seven forms of struggle narratives.

## Introduction

Newspapers are the oldest mass media that play an important role in disseminating information. ^1^ As a powerful means of collective communication, the mass media makes it easy for people to keep getting the most up-to-date information around them every day. Media images formed in the mass media can influence aspects of human life personally and professionally. ^2^ In national politics, the mass media is the most fundamental communication tool for nation-building, national integration and national identity. ^3^ Through the right image media, can communicate a nation’s political ideas and goals to its people to strengthen nation-building, national integration and national identity.

In addition, the mass media can also facilitate the formation of national commitment and national unity. Based on three perspectives: 1) standardizing and uniting national discourse in the same language; 2) providing an image and point of view of the existence of a nation in international relations; 3) strengthening social relations within the nation itself through various news featuring similarities in culture, history, language, traditions, religion, symbols, destiny and a sense of belonging to one another. ^4^ Based on this description, can observe that newspapers play an important role in fostering a sense of nationalism, instilling the concept of “nation”, fostering a sense of belonging and maintaining unity. The colonized nation urgently needs this spirit of nationalism and unity to achieve independence and build a self-sovereign government.

Many previous studies found the newspapers’ contribution to the colonised nation’s independence. Derderian ^5^ finds the role of newspapers as forming the concept of nation and colonialism in 19 ^th^-century Armenia. Gan ^6^ in his research on The May 30 movement in Shanghai in 1925, found that newspapers were a medium of communication for people’s struggles. In addition, newspapers used as a medium to said the nationalism of the Shanghai people as well as sides of politics. For over a century Scottish peoples expressed nationalism in the regional press in their struggle for independence from England. ^7^ Meanwhile, Doyle ^8^ found that newspapers were a propaganda tool in forming the modern nation-state during the independence movement in Ireland.

Kirubakaran’s research ^9^ on India’s struggle for independence from Britain, found that Indian-language newspapers were the front line in India’s struggle for independence. Newspapers in the Indian language carried the news “message of rebellion to the nation” published every week. Jain ^10^ finds messages of rebellion conveyed in Indian language newspapers during the War of Independence. The news content was radical and bluntly called on all Indian people to support the ongoing struggle for independence. Indian newspapers gave colossal support to radical groups and extremist leaders in the form of publishing news stories that justify all the actions of these extreme and extremist groups for India’s independence. Indian newspapers also encouraged the people to carry out the *Swadeshi* movement by boycotting British products, carrying out strikes at British companies, and using products made in India. ^11^

The research from various countries above shows that indigenous newspapers played an important role as a tool in the struggle for independence from colonialism. The fight for independence in Indonesia began with a national movement spearheaded by indigenous educated groups in the early 1900s. The political improvement of the Indonesian movement is correlated with the progress of the print press media which was used as the spearhead of the struggle. Newspapers were introduced by the Dutch Colonial Government starting in 1659 for their benefit, adopted, practised and developed by Indonesian educated people. Indigenous intellectuals who initially became newspaper readers eventually switched to becoming newspaper writers and publishers. They wrote various thoughts on nationalism, the importance of unity as a nation, and the right to independence for the Indonesian people. These educated people also used newspapers as a medium of communication between themselves in spreading the ideas of nationalism, unity and the goal of independence they fought for.

North Sumatra became one of Indonesia’s newspaper publishing centers during the Dutch colonial period. This evidenced by the number of newspapers published in this region from 1900-1942, reaching more than one hundred and forty newspaper names. ^12^ These newspapers have content and reporting themes representing various groups and interests. This study aims to investigate the involvement of indigenous newspapers in the struggle for Indonesian independence in North Sumatra during the Dutch colonial period and highlight how narrated the contents of the newspaper’s coverage in the struggle for Indonesian independence. To achieve this goal, will answer the following research questions.

RQ1: How many indigenous newspapers in North Sumatra during the Dutch colonial period supported the struggle for Indonesian independence?

RQ2: How is the representation of the struggle for Indonesian independence narrated in the newspaper article?

## Methods

This study uses a qualitative content analysis (QCA) method, which aims to describe, analyze, and interpret the meaning of textual data systematically. ^13^^–^^16^ Qualitative content analysis does not only explore the surface of the text by counting words or examining hidden meanings, themes and patterns but goes deeper into the researcher’s subjective understanding of social reality in a scientific way. ^17^

Understanding or subjective interpretation of the text’s content is obtained through coding and identifying themes or patterns. ^18^ The coding of the unit of analysis in the qualitative content analysis is more varied because it adjusted to the needs of the analysis. ^19^ Qualitative content analysis has two forms: the description of manifest content (frequency) and the description of latent content (meaning). ^20^^,^^21^ If the two types of analysis are combined, the results of the qualitative content analysis will be more in-depth. ^22^

The procedures and techniques for conducting qualitative content analysis follow a qualitative systematic review that produces data in words and themes that can interpreted. ^23^^,^^24^ Textual material that is the focus of study from qualitative content analysis includes interview transcripts, transcripts of focus groups, textbooks, company brochures, contracts, diaries, newspaper articles, magazine advertisements, websites, email messages, letters, and many more. ^19^^,^^23^

**Figure 1.**
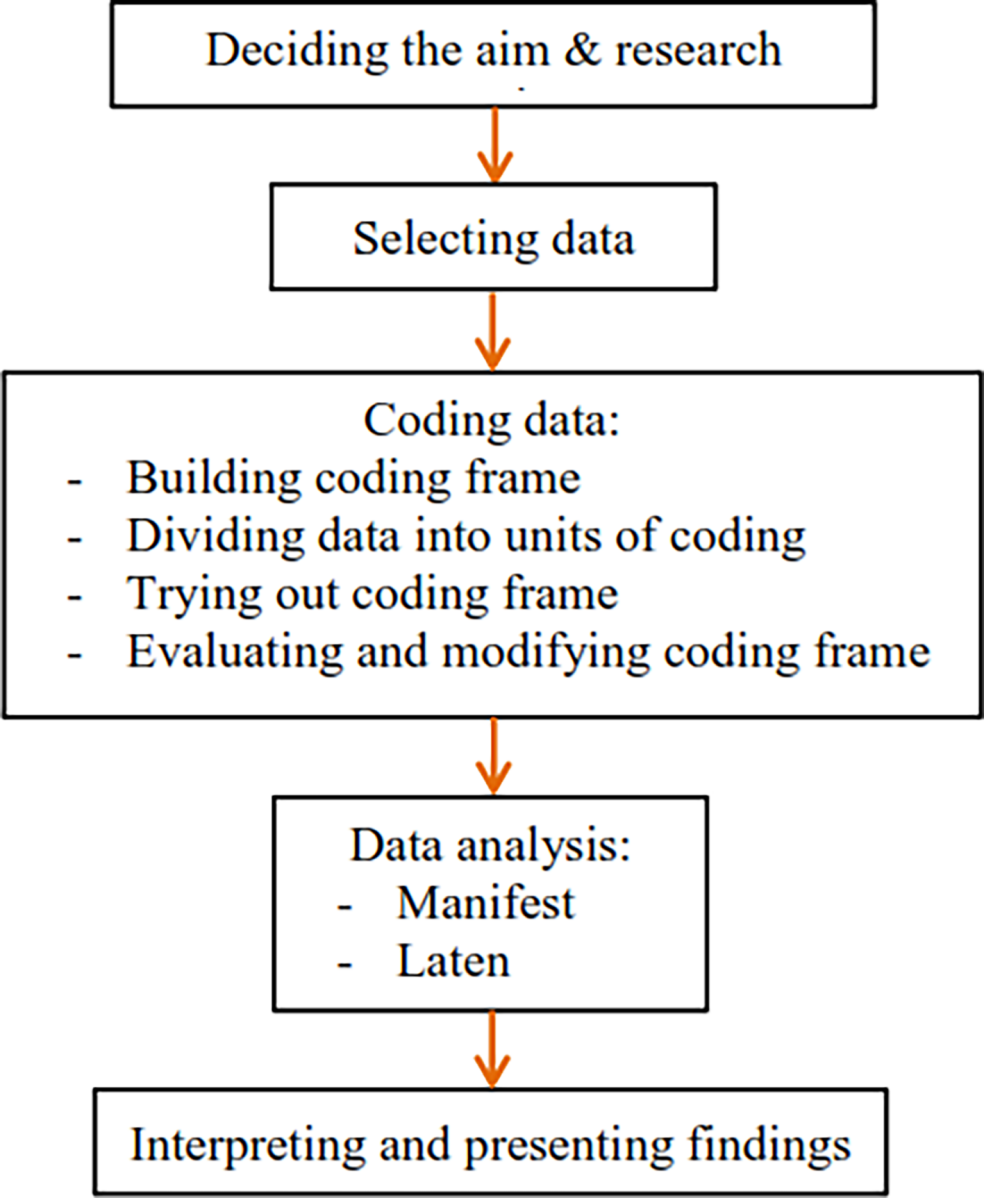
Research design.

This research scheme uses the Schreier ^23^ design method, which consists of eight stages: 1) Deciding the aims and research questions; 2) selecting data; 3) building coding frames; 4) dividing data into units of coding; 5) trying out coding frames; 6) evaluating and modifying coding frames; 7) Data analysis; 8) interpreting and presenting findings. ^23^ In Figure 1, it can be observed how carried out the procedures in this study from start to finish.

### Selecting data

The data used in this study were printed newspapers published in North Sumatra during the Dutch colonial period. These newspapers are about 100 years old, do not yet have a digital version, and not all editions are in complete condition. In addition, parts of the papers are damaged and letters that need to be clarified meaning that some of the writing cannot be read. This factor told that the research carried out very carefully not to hurt ancient texts, categorized as historical artifacts and optimal in text content analysis.

A good sample is the most important indicator that must prepared in the research process. ^25^ The sample of this research is printed newspapers published by indigenous people in North Sumatra during the Dutch colonial period. In content analysis, the determination of the sample must be based on the unit to be analyzed. ^19^ Research samples in qualitative content analysis are usually consistently selected texts purposively selected or to answer research questions. ^17^ Relevance sampling or purposive sampling techniques involve all textual units and follow a conceptual hierarchy, which reduces the number of units to considered for analysis. Researchers have full rights to determine the units specified as a sample. ^26^^–^^29^ As shown in Figure 1, selected the data for this study after deciding the aim and research questions. The research purposes and questions in the introduction section are used as the unit of analysis to determine the research sample. Based on the selected data that has been collected, the research sample in this study is indigenous newspapers that publish news of the struggle for Indonesian independence in North Sumatra during the Dutch colonial period.

The data selection and sampling process lasted two months (May to June 2020). Sample collection was carried out at three research sites that housed collections of Indonesian newspapers from the Dutch colonial period, namely the National Library of Indonesia, the Medan History House, and the Center for the Study of History and Social Sciences, Universitas Negeri Medan (Pussis-Unimed). This process began with searching online library catalogues and offline library catalogues in the National Library of Indonesia (OPAC), offline library catalogues in Medan History House and Center for the Study of History and Social Sciences, Universitas Negeri Medan (Pussis-Unimed). After the data regarding the names of the newspapers obtained from the catalogues, the next step was to read them one by one, check them one by one and calculate all the newspaper data found at the three research sites. This is done to find the topic or content of each article.

Reading newspaper articles was carried out carefully due to differences in language style and diction in newspapers published during the Dutch colonial period with the current Indonesian style to avoid misinterpretation. The topic of each article then linked to the selection guidelines “Indonesian independence news and Indonesian independence propaganda”. Articles related to the selection guidelines were contained in the sample category, while those not described in the non-sample category will be included. The data included in the sample category were then transferred into written text in Microsoft Word to proceed to the coding stage.

### Coding process

After selecting data, the coding process or material coding consists of four stages: building the coding frame, dividing data into coding units, trying out the coding frame, and evaluating and modifying the coding frame. Preparing a coding scheme or coding frame is the initial stage of the coding process in qualitative content analysis. ^18^^,^^23^^,^^30^ The preparation of the coding scheme must be objective and reliable. ^29^ The purpose of compiling a coding frame or scheme is to structure, classify and simplify sample units or data into a category for easy analysis by researchers. ^23^^,^^31^^,^^32^ In qualitative content analysis, categories or dimensions can be developed from available data or material (without being based on theory), research focus, or research questions. ^30^^,^^32^ The coding frame of this study is arranged according to the categories of De Hert *et al.* (2023), which adjusted to the research objectives and the results of data selection. ^33^

**Figure 2.**
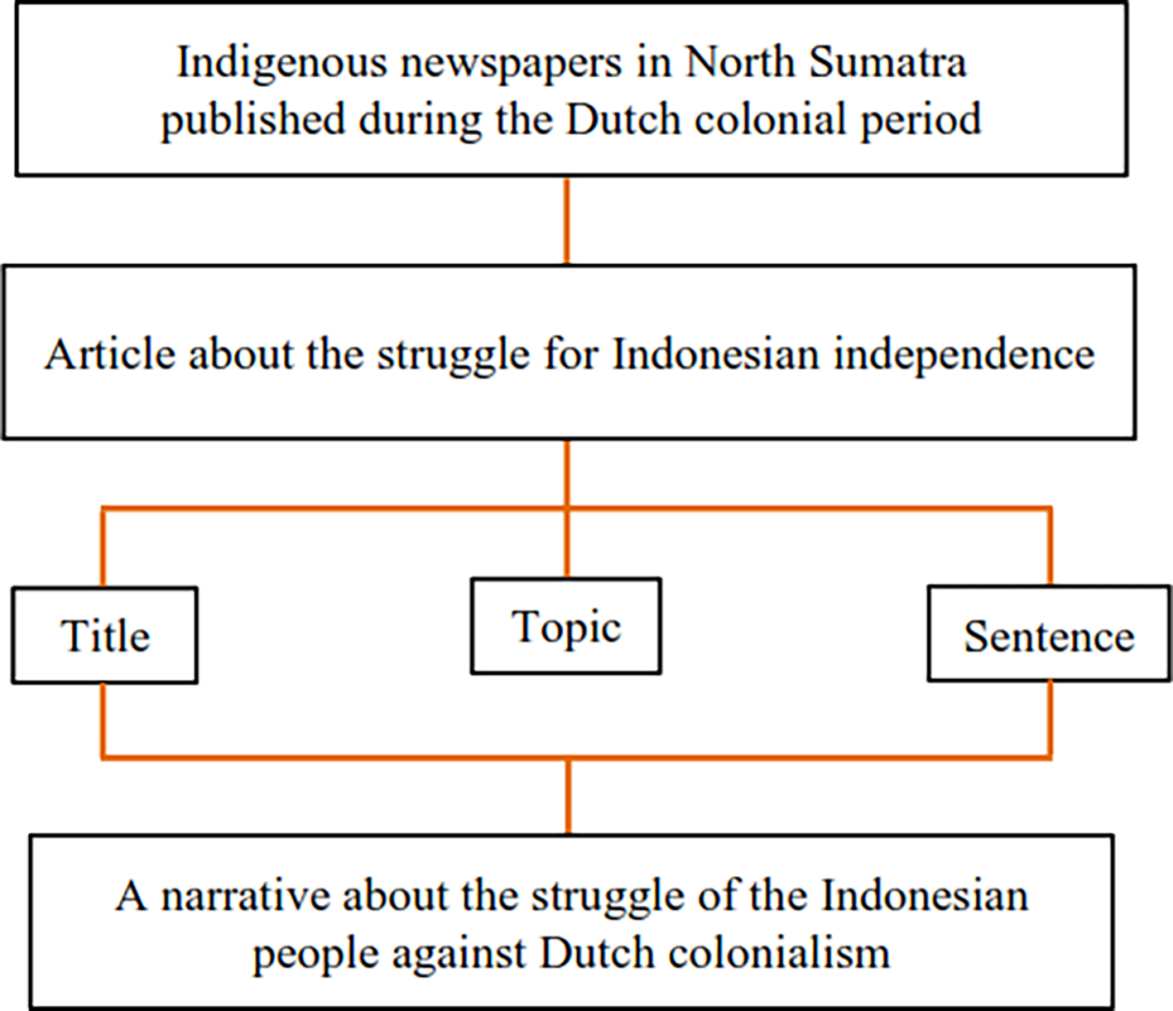
Coding frame.

The data obtained in selecting data is then divided into coding units (in the form of tables) in Figure 2. The following process is trying out the coding frame. Two graduate students were trained as coders for one week. Coders were instructed to analyze the data in the coding unit table and put a checkmark in the column provided. The results of the trying-out coding form are then evaluated and modified by considering coders’ suggestions. The repaired coding frame is then tested again on the same coders.

The validity test was conducted by peer-reviewing coding books and coding forms by journalistic and media experts from FJPI North Sumatra. The reliability test was based on intercoder reliability involving coders around the clock. ^34^ Intercoder reliability requires a minimum of two independent coders, as used in this study. ^35^ The reliability test for this study uses NVivo interrater reliability, including the Percent agreement, Scott’s pie (π) and Cohen’s kappa. ^29^^,^^36^^,^^37^ The reliability test results obtained PAo = 89% (N = 107), Holsti method Pi = 0.88. Cohen’s kappa = 0.88. Based on statistical ranges from 0.00 (no agreement) to 1.00 (perfect agreement), the three reliability agreements are at a strong (high) level.

### Data analysis

The analysis in this study was carried out in two stages, 1) manifest analysis and 2) latent analysis. The combination of manifest and latent content aims to analyze the meaning that appears on the surface of the text and the hidden meaning in the newspaper text under study. The manifest analysis focus on the structure or surface part of the text that can be seen, observed and counted. ^13^^,^^19^^,^^24^ The surface part of this text is limited to the transcription or the original form written on the text in the form of the language and words used or the topic of the text. ^19^^,^^29^^,^^31^^,^^38^ The latent analysis aims to find hidden meanings or ideas in the structure of the text that are not visible on the surface to find the real message the writer wants to convey to the reader. The latent analysis was applied by closely reading the text and coding frame, guided by the research questions. ^19^^,^^24^^,^^29^^,^^31^^,^^23^^,^^39^ In this study, manifest analysis was carried out on the categories name of the newspaper, publication date, the title of the article, the topic of the article, and sentences in the text. The latent analysis appears on the article’s topic and sentences in the text. The results of the analysis are then tabulated and interpreted. The next stage is drawing conclusions displayed in tables, charts and descriptive descriptions.

## Results

### The indigenous newspapers in North Sumatra that supported Indonesia’s struggle for Independence from 1916-1925

This research found 107 articles in 13 newspapers supporting the Indonesian Independence struggle during the Dutch colonial period. Table 1 displays the names of the 13 indigenous newspapers published in North Sumatra, the year of publication, and the number of articles in each newspaper. In Table 1 can observe that *Soeara Batak* is the newspaper with the most articles, namely 20.56% (22 articles). *Sinar Zaman* and *Orgaan Bataksche Studiefonds* are the newspapers with the least publications, namely 0.93% (1 article each). In Table 1, it can be seen that Medan is the city that publishes the most indigenous newspapers, with a total of 9 newspapers. Kota Nopan, Tanjung Balai, Binjai and Tarutung only have one newspaper each, *Orgaan Bataksche Studiefonds*, *Al Moektabas*, *Tjermin Karo*, and *Soeara Batak.*

**Table 1.** Number of indigenous newspaper articles in North Sumatra (1916-1925).

No	Newspaper name	Publication date	City of publication	Number of articles	%
1.	*Soeara Djawa*	6/1/1916 – 7/1/1918	Medan	9	8.4
2.	*Pewarta Deli*	7/2/1917 – 8/1/1917	Medan	5	4.6
3.	*Benih Merdeka*	7/2/1918 – 4/8/1920	Medan	12	11.21
4.	*Perempoean Bergerak*	5/15/1919 – 12/15/1920	Medan	11	10.28
5.	*Soeara Bondjol*	6/1/1920 – 8/1/1922	Medan	3	2.80
6.	*Sinar Zaman*	10/8/1921	Medan	1	0.93
7.	*Orgaan Bataksche Studiefonds*	2/1/1921 – 3/31/1922	Kota Nopan	1	0.93
8.	*Andalas*	11/1/1923 – 11/6/1923	Medan	5	4.67
9.	*Mandailing*	12/13/1923 – 3/3/1923	Medan	12	11.21
10.	*Warta Timur*	10/8/1923 – 11/29/1923	Medan	13	12.15
11.	*Al Moektabas*	1/31/1924	Tanjung Balai	6	5.61
12.	*Tjermin Karo*	1/13/1925 – 12/28/1924	Binjai	7	6.54
13.	*Soeara Batak*	2/21/1920 – 12/24/1925	Tarutung	22	20.56
Total				**107**	**100**

In Table 2, it can be observed that 1923 was the year that published the most news on the struggle for Indonesian Independence, with a total percentage of 31.78% by five newspapers, namely *Soeara Bondjol*, *Andalas*, *Warta Timur*, *Mandailing*, *Soeara Batak.* 1916 was the year with the fewest, namely 1.87%, published by *Soeara Djawa* (see Table 2).

**Table 2.** Distribution of years of publication of indigenous newspapers in North Sumatra (1916-1925).

Tahun	Newspaper name	Number of articles	%
1916	*Soeara Djawa*	2 articles	1.87
1917	*Pewarta Deli*	5 articles	4.67
1918	*Soeara Djawa* *Benih Merdeka*	7 articles 6 articles	12.15
1919	*Perempoean Bergerak*	5 articles	4.67
1920	*Benih Merdeka* *Perempoean Bergerak* *Soeara Bondjol* *Tjermin Karo* *Soeara Batak*	6 articles 6 articles 1 article 1 article 6 articles	18.69
1921	*Sinar Zaman* *Orgaan Bataksche Studiefonds* *Soeara Batak*	1 article 1 article 4 articles	5.61
1922	*Soeara Bondjol* *Soeara Batak*	1 article 2 articles	2.80
1923	*Soeara Bondjol* *Andalas* *Warta Timur* *Mandailing* *Soeara Batak*	1 article 5 articles 13 articles 12 articles 1 article	29.91
1924	*Al Moektabas* *Tjermin Karo*	6 articles 5 articles	10.28
1925	*Tjermin Karo* *Soeara Batak*	1 article 9 articles	9.35
Total		**107**	**100**

### The narrative of the struggle for Indonesian Independence in the indigenous newspapers published in North Sumatra from 1916-1925

Manifest analysis and latent analysis on titles, topics and sentences found 7 narrative forms of the struggle for Indonesian Independence found in indigenous newspapers in North Sumatra from 1916-1925. The seven narrative forms are: 1) demanding Indonesian Independence, 2) national movement, 3) nation-building, 4) unity, 5) patriotism, 6) criticizing Dutch colonialism, and 7) the misery of the people of North Sumatra. The seven narratives were identified through analysis of the title of the articles, the topic of the article, and the sentences contained in the report. Some of these narratives written by extrinsically and intrinsically.

In Table 3, it can be observed that the National Movement is the most common narrative found in the analysis of titles, topics and sentences in articles. 36.73% was found in the title (N = 49), 26.09% in the topic (N = 89), and 23.72% in the sentences within the text (N = 156). Criticism and demands to the Netherlands were the least reported narrative in the analysis of article titles, with a percentage of 2.04%. In the topic analysis, patriotism is the least prevalent in the record, with a rate of 5.43%. The patriotism narrative is also the least found in the study of sentences in the text by 6.41% (see Table 3).

**Table 3.** The results of the analysis on the title of the article, the topic of the article and the excerpt of the sentence.

Article category	Article title		Article topic		Sentences in the text	
	Number of articles (N=49)	%	Number of articles (N=92)	%	Number of articles (N=156)	%
Demanding Indonesian Independence	6	12.24	12	13.04	20	12.82
National movement	18	36.73	24	26.09	37	23.72
Nation-building	14	28.57	12	13.04	30	19.23
Unity	3	6.12	10	10.87	25	16.03
Patriotism	2	4.08	5	5.43	10	6.41
Criticism and demands on the Dutch colonial	1	2.04	10	10.87	15	9.62
People's misery	5	10.20	19	20.65	19	12.17

Examples of narratives from the National Movement can be found in titles such as “The Age of Progress”, “Our Movement” and “World Movement of the Mandailing People” (see *Underlying data*). The narrative of the National Movement found in the topic of the articles includes “Appeals to support the Age of the movement”, “Establishment of *werklub* for economic progress and Indonesian independence”, “The indigenous people must rise against injustice and misery due to colonialism” (see *Underlying data*). The narrative of the National Movement found in the sentences in the text is in the form of “Together we support this emerging movement”, and “Meant going directly against the existing power” (see *Underlying data*).

The narrative of criticism and demands on the Dutch was founded in the title of the article “Want to be noticed by the government”. The description of patriotism is found in the topic of the article “The Cry of Love for the Nation and the Motherland” “How to Love the Motherland”. In the sentence in the article, the narrative of patriotism was founded in “Homeland is where our nation’s blood spilt”, “Since the birth of the people’s movement in Indonesia the words “Love the nation and the motherland” are often listed in newspapers and meeting" (see https://doi.org/10.6084/m9.figshare.23796597).

## Discussion

In the history of various countries that succeeded in becoming independent from colonial rule, indigenous newspapers have played an essential role in supporting the struggle for independence. The pen is a sharp weapon, and the newspaper is a revolutionary instrument. ^40^^–^^42^ In Indonesia, the emergence of the press in the fight for independence was in line with the political dynamics that occurred, especially during the rise of Indonesian nationalism, which gave rise to Indonesian consciousness which became the starting point of the struggle in the first decades of the early 20 ^th^ century. ^43^^,^^44^ The increased political activity of the Indonesian independence movement against the Dutch contributed to the growth of the circulation of native newspapers, especially in the second and third decades (1910s-1920s), known as the radical political period. Newspapers at this time were used to advocate Indonesian freedom through narratives written and published to the broader community.

This research finds seven categories of narratives that support the struggle for Indonesian independence in indigenous newspapers published in North Sumatra from 1916-1925. The seven narrative categories are 1) demanding Indonesian Independence, 2) national movement, 3) nation-building, 4) unity, 5) patriotism, 6) criticizing Dutch colonialism, and 7) the misery of the people of North Sumatra. The findings of the seven narratives which represent the role of the press as a tool for the struggle for independence during the period of the Indonesian National Movement were founded by Said, ^12^ Adam, ^43^ Abdullah, ^45^ Juliati & Asnan, ^46^ Reid, ^47^ Sumanti *et al.*, ^48^ Azhari. ^39^ But unfortunately, the previous research did not elaborate on the form and type of narrative from each article as was done in this research.

Based on the description in the results section, national movement was the most common narrative found: 36.73 % (title), 26.09 % (topic), and 23.72 % (sentences in the text). There was a large number of articles that wrote about the national movement because 1916-1925 was the national movement period in Indonesia. This period of the national movement gave rise to a heightened awareness among the people and the ambition to achieve progress on their own. ^49^ The indigenous press became a medium for the expression of Indonesian nationalism. ^46^ Medan’s position as the centre of the anti-colonial movement made this city the centre of the struggle of political figures from the North Sumatran movement, such as Mohammad Samin, Mohamad Yunus, Abdul Muis. ^44^ This movement figure made newspapers the spearhead against Dutch colonialism. From the research findings, 9 out of 13 North Sumatran indigenous newspapers were published in Medan (see Table 1). If we observe the national movement in India, the relationship that was formed between movement figures and newspapers also occurs, in which Indian political leaders use newspapers as a tool to build people’s nationalism and popularize the *Swadeshi* movement. ^9^^,^^11^ In the American Revolution, political readers made newspapers a medium for spreading revolutionary rhetoric, literacy, civic discourse and political dissent. ^50^

Although the national movement narrative is the most commonly obtained finding, the narrative “Demanding Indonesian independence” is the most important point of this research finding. This is because “independence” is the main goal of what the Indonesian people want as a colonized country. Independence became the culmination of the national movement that the colonized people fought for. The struggle for independence involving the role of newspapers also occurred in the Scottish independence struggle, which is still ongoing today. ^7^ The findings of the narratives of demands for independence published in native newspapers in North Sumatra in 1916-1925 were in the form of open calls for the Dutch to grant independence, open appeals to the people to fight for independence, and implied demands for independence. The narrative of demands for independence is found in the *Soeara Batak* edition of January 27, 1923, with the article titled “Is it true that Indonesia will soon be given independence by the Dutch colonial?” An article containing an appeal to the people to fight for Indonesian independence was published in the May 29, 1918 edition of *Benih Merdeka* with the title “Freeing the Nation”, the August 27, 1921 edition of *Soeara Batak* with the article title “Achieve independence”, the January 27, 1923, edition of *Mandailing* with the article title “Faith and independence”. Implicit demands for independence were contained in *Benih Merdeka* with the article title Remember Fate!” (May 30, 1918), *Soeara Djawa* “Movement” (June 1, 1918), and “We are responsible! We indict!” *Benih Merdeka* (16 March 1920) (see https://doi.org/10.6084/m9.figshare.23796597).

The implied meaning of the demand for Indonesian independence can be identified from the sentence quotes listed in the article. In the article entitled “Remember Fate!!” are the words “Indonesia is free and can be independent” and “Free Indonesian land”. The article “Movement” states, “It is necessary to establish a *Werklub* based on association and evolution to advance Indonesia’s economy and independence”. In the article entitled “We are responsible! We indict!” is the sentence “An alliance that intends to establish a native authority itself”. The sentences above, although they do not mention Indonesia’s Independence from Dutch colonialism, use of words like free, independent, independent Indonesia, and indigenous power itself are implied expressions of the struggle being carried out by the elite national movement to spread the spirit of independence to newspaper readers and the wide community. The implied expression also aims to ask for the support of the people of North Sumatra to support the ongoing struggle so that Indonesian Independence can be realized. Through newspapers, the concept and model of nationalism spread quickly, especially among indigenous people who have been able to publish their newspapers. ^51^

National movements and demands for independence will only exist with being preceded by nation-building. This is because nation-building is a mass consolidation effort that encourages citizens or members of a nation to have the same identity, goals, perceptions, and interests of the nation and their homeland so that they can unite and not be divided from one another. ^52^^,^^53^ The mass media has a very fundamental role in nation-building and integration. ^3^ The origins of national consciousness in colonized countries cannot be separated from the role of printed newspapers. ^51^ In Indonesia, national awareness was the initial goal of the emergence of indigenous newspapers, including in North Sumatra. ^45^ In its development, newspapers became the most effective propaganda tool in voicing the people’s desire for independence. ^54^

Criticism and demands for the Dutch and patriotism were the fewest narrations found, especially for narrations in the text titles. This happened because of the censorship system imposed by the Dutch colonial government through the rules of the *Koninklijk Besluit* (king’s decree) dated March 16, 1906, which required printers and publishers to submit one copy to the local regional government within 24 hours of circulation. ^46^ This rule made the authors of articles in newspapers very careful in presenting narratives in article titles, especially those that criticized and denounced the practice of Dutch colonialism in North Sumatra. The act of caution in writing the title of this article is very acceptable if we compare it with the results of the analysis of topic articles, where narratives of criticism and demands for the Netherlands are the second most common article (20.65%) after the national movement (see Table 3).

The results of the analysis also found multiple meanings in an article, such as the double meaning between nation-building/unity/patriotism found in the sentences in the article. This happens because nation-building, which aims for a collective imagination of belonging and the process of exacting a national identity, will always be related to unity and patriotism. ^55^ This narrative, which has a double meaning, includes: “Allowing and giving opportunities to various nations to advance their homeland and nation” ( *Al Moektabas*) and “O my people whom I love, remember! If we don’t immediately unite our minds, agree to achieve common progress” ( *Soara Batak*) (see https://doi.org/10.6084/m9.figshare.23796597). The diversity of narratives in one sentence is also found in the article “Movement” published in *Soeara Djawa.* This article narrates the demand for Indonesian independence and unity among the natives. In the article entitled “We are responsible! We indict! published in the *Benih Merdeka* newspaper also found the same narrative regarding the demand for Indonesian independence and the unity of the indigenous people.

The role of indigenous newspapers in North Sumatra in the struggle for Indonesian Independence was increasingly crucial due to supervision and pressure from the Dutch colonial government. The courage of the indigenous newspapers in demanding Indonesian independence was seen as a threat to the stability of Dutch rule in their colonies. Therefore, the Dutch Colonial Government established rules for publishing newspapers which were published in the *Strafwetboek* (Book of Criminal Law). In article 66a *Strafwetboek* it is stated that anyone who spreads hostility, hatred or contempt for the Dutch government or the Dutch East Indies, either through writing, pictures or actions, will be sentenced to 5-10 years in prison. Meanwhile, in article 66b *Strafwetboek* it is stated that anyone who spreads hostility, hatred or insults among the population of the Dutch East Indies, either through writing, pictures or actions, will be punished with imprisonment for 6 days to 5 years. ^44^

The regulations for publishing the print press at Strafwetboek did not make North Sumatran indigenous press figures afraid of making newspapers their tool of struggle. This courage was proven by publishing articles on the theme of nationalism in the June 1, 1916 edition of the *Soara Djawa* newspaper. In an article entitled “Pen turned to the esteemed brother” the author asks all Indonesian people to support the ongoing movement (see https://doi.org/10.6084/m9.figshare.23796597). The movement referred to in this newspaper is progress which is interpreted as an effort to improve the lives of the Indonesian people in the economic, social, cultural and political fields so that they are no longer inferior and colonized. The progress made was intended so that the Indonesian people could liberate themselves from Dutch colonialism, which was very miserable. ^44^

## Conclusion

North Sumatra became one of the areas of Indonesia’s struggle for independence, making indigenous newspapers a means of struggle to liberate themselves from Dutch colonialism. Indigenous newspapers became the most important media to strengthen nation-building, national integration and national identity among the colonized people. The role of newspapers in the struggle for independence in North Sumatra is evidenced by the findings of 107 articles in 13 newspapers in seven forms of struggle narratives: 1) demanding Indonesian independence, 2) national movement, 3) nation-building, 4) unity, 5) patriotism, 6) criticizing Dutch colonialism, 7) the misery of the people of North Sumatra. The seven narrations are part of the article title, article topic, and sentences in the article were written extrinsically and intrinsically.

The national movement is the most commonly found narrative in research. In the analysis of the title, found as many as 18 articles. The topic analysis found 24 articles and sentence analysis found 37 articles. Demanding Indonesian independence is the most important narrative, although the number is less than that of the national movement and nation-building. The findings of the narratives demanding Indonesian independence are evidence of the role of indigenous newspapers in North Sumatra in the struggle for Indonesian Independence during the Dutch colonial period. The figures of the North Sumatra independence movement fearlessly demanded Indonesian independence and called on the people of North Sumatra to fight for the liberation of their nation from Dutch colonialism through the narratives they wrote in the newspapers. Even though the Dutch Colonial Government would give punishment for their act of resistance, this in no way dampened their struggle to spread narratives in newspapers for the sake of Indonesian Independence.

## Data availability

### Underlying data

The sources of newspapers in our research exist and can be referred to by other researchers at the Jakarta National Library using their website https://www.perpusnas.go.id/ or by contacting the following email: materjilperpusnas20@gmail.com. The librarian on duty will answer the email sent to this email address. In addition, these sources can also be seen (offline access only) at the North Sumatra Press Museum, Sei Alas Road No. 6, Medan. The Medan History House, Kota Cina/Pematang Siombak Road, No. 65, Neighborhood 7, Paya Pasir Village, Medan Marelan District, Medan 20250. The Center for the Study of History and Social Sciences Universitas Negeri Medan (Pussis-Unimed), Willem Iskandar Road, Pasar V, Medan Estate.

Figshare: ‘The analysis table of titles, topics, and sentences in indigenous newspapers in North Sumatra during the colonial period’ [ https://doi.org/10.6084/m9.figshare.23796597]

• The title of the article which wrote about the struggle for Indonesian independence in indigenous newspapers in North Sumatra (1916-1925).
• The results of the analysis of the topic of the article.
• The results of the analysis of sentences written in the article.

### Extended data

Figshare: Table of indigenous newspapers published in North Sumatra in 1916-1925 10.6084/m9.figshare.17894687

Data are available under the terms of the Creative Commons Attribution 4.0 International license (CC-BY 4.0).
