# Supplementary material for: The role of newspapers published in North Sumatra during Indonesia's independence struggle between 1916-1925: A content analysis
Source: F1000Res. 2023 Oct 6;11:249. Originally published 2022 Feb 28. [Version 2] doi: 10.12688/f1000research.53442.2 (PMC9478501; doi:10.12688/f1000research.53442.2)
Supplement: Peer review history

# Reviewer report: Reviewer response for version 1

**DOI:** 10.5256/f1000research.56826.r149741

**Published:** 2022-09-21

**Authors:** Kwabena Sarfo Sarfo-Kantankah

**Affiliations:** 1 University of Cape Coast, Cape Coast, Ghana

**Summary**

 Using newspaper articles as data and a critical discourse analytical approach, the paper attempts to demonstrate the role newspapers played in Indonesia's struggle for independence from 1916 to 1925. The paper shows that out of the 24 newspapers that were published in North Sumatra, the centre of newspaper publications in Indonesia, 13 published articles that concerned the quest for independence by Indonesia, including, 1) demanding Indonesian independence openly; 2) criticizing various policies of the Dutch Colonial Government; 3) building awareness of Indonesian nationalism. 

**Is the work clearly and accurately presented and does it cite the current literature?**

 The paper is written in clear, simple, and straightforward language. It makes easy reading. However, it hardly cites current literature. The authors' engagement with literature, especially in the analysis and discussion, is very low. This makes it difficult to situate the analysis and discussion in the context of current literature so as to measure the extent of the paper's contribution to scholarship. 

**Is the study design appropriate and is the work technically sound?**

 The authors state that the paper uses a qualitative research design, which is appropriate. However, they fail to state the specific qualitative design employed, even though it is clear that the paper does a content analysis. The authors should therefore consider content analysis as the design used and describe how they deployed it. The work is technically sound, except for the issue I have raised.

**Are sufficient details of methods and analysis provided to allow replication by others?**

   

 The methods section is not detailed enough to allow for replication. The authors indicate that they employ the critical discourse analysis (CDA) method, however, they do not clearly indicate how CDA informs the work. Their discussion of CDA is too general; CDA should be clearly defined and the authors should be specific about how they deploy methods of CDA.

 The authors fail to systematically delineate how the data were gathered, processed, and analysed. For example, how did they assemble the data? Did they manually read the newspaper articles to identify specific instances of words, phrases, clauses, sentences, and other structures of interest? A statement such as " *The first step of data analysis is to read one by one the articles from 24 indigenous newspapers in North Sumatra in Table 1. This first stage uses macrostructural analysis to find topics or themes contained in a text*" should appear under methods, where the authors systematically describe the methods of data analysis. 

 The analysis and discussion appear too superficial: the authors do not analyse specific text samples (sentences, clauses, etc.) to tease out "hidden" meanings or macro issues (e.g. power, struggle) as it is usually done by CDAs. The paper appears to concentrate too much on counting instances of titles and expressions/sentences/words rather than discussing and arguing about the discourse in the newspapers, thus, making the claim that the paper does a CDA quite weak. In other words, there is little or no textual analysis. For example, under the Discussion, the authors state: " *Microstructural analysis based on the meaning of words and sentences emphasized in the text, the form and structure of the sentences presented, the choice of words used, and the expression or style of language explicitly shows the demand for Indonesian independence in twelve articles with this theme*". However, I do not see any analysis of the form and structure of sentences – the authors just list sentences/phrases, without discussing their forms and structures. 

**Are the conclusions drawn adequately supported by the results?**

 While their conclusion, as it stands, is drawn partly supported by the results, it is weak as a result of the issues I have raised above. 

 In the last paragraph of the Discussion section, the authors state: “ *The above description shows that the print media is the most important element in shaping the concept of nationalism of a nation*.” The statement seems too general; it should be limited to the Indonesian context. 

**Other issues**

 The authors should do some language editing. There are several language infelicities. Some of them are indicated below. 

- *"There are twelve articles in four newspapers **which** ****openly wrote about the demands for Indonesian independence."*
- *"In the Pewarta Deli newspaper entitled “Indies Nederland” the **sentence** **[phrase, not a sentence]** “Arbitrary acts of Dutch officials” was written."*
- *"The people of North Sumatra **use [used] **newspapers as a tool for the struggle for Indonesian independence to the fullest through 51 articles found in this study."*
- *"The most important thing about publishing this newspaper is the role of **newspapers [paper?]** as a tool for the struggle for independence against Dutch colonialism."*
- *"People in North Sumatra **use [used]** newspapers as a tool for the struggle for independence as much as possible."*

Is the work clearly and accurately presented and does it cite the current literature?

Partly

If applicable, is the statistical analysis and its interpretation appropriate?

Not applicable

Are all the source data underlying the results available to ensure full reproducibility?

Yes

Is the study design appropriate and is the work technically sound?

Partly

Are the conclusions drawn adequately supported by the results?

Partly

Are sufficient details of methods and analysis provided to allow replication by others?

Partly

Reviewer Expertise:

(Critical) Discourse Analysis, Pragmatics, Corpus Linguistics

I confirm that I have read this submission and believe that I have an appropriate level of expertise to confirm that it is of an acceptable scientific standard, however I have significant reservations, as outlined above.

# Reviewer report: Reviewer response for version 1

**DOI:** 10.5256/f1000research.56826.r145899

**Published:** 2022-09-15

**Authors:** Hafizah Hajimia

**Affiliations:** 1 Department of English and Linguistics, Academy of Language Studies,, Universiti Teknologi MARA, Arau, Malaysia

The paper was well written. However, I have a few comments.

**Comments**:

- Verification of the result is essential to show the trustworthiness of the findings. How were the findings analysed? How was the data verified?
- Lack of elaboration in the methodology section. More elaboration was given on the CDA.
- Most references have exceeded 5 years.

**Suggestions:**

- Need to add more elaboration in the methodology section especially the data analysis method, reliability of the findings, and tools used to analyse the data.
- Elaborate on how did you obtain the numbers in the tables of your results. What tools and methods did you use to derive the numbers?
- Elaborate more on how you conducted the superstructure analysis, microstructural analysis, and the other stages, steps, tools, and procedures.
- To reduce bias, validity and reliability of the findings need to be included.
- Would be good if some current references are included.

Is the work clearly and accurately presented and does it cite the current literature?

Partly

If applicable, is the statistical analysis and its interpretation appropriate?

Yes

Are all the source data underlying the results available to ensure full reproducibility?

Partly

Is the study design appropriate and is the work technically sound?

Yes

Are the conclusions drawn adequately supported by the results?

Partly

Are sufficient details of methods and analysis provided to allow replication by others?

No

Reviewer Expertise:

Applied Linguistics, TESL, Education, Corpus Linguistics, Discourse analysis, Genre analysis, English for Specific Purposes

I confirm that I have read this submission and believe that I have an appropriate level of expertise to confirm that it is of an acceptable scientific standard, however I have significant reservations, as outlined above.

# Reviewer report: Reviewer response for version 1

**DOI:** 10.5256/f1000research.56826.r145900

**Published:** 2022-09-12

**Authors:** Jane Nkechi Ifechelobi

**Affiliations:** 1 Department of English, Nnamdi Azikiwe University, Awka, Nigeria

The article claimed to have used CDA as the method applied in this research but areas such as power, dominance and nationalism were not reflected on. The authors should look at the first paragraph of the methods and approach the work appropriately.

 The authors conducted a content analysis. I suggest the article title is changed to reflect this, e.g. "An analysis of…".

 The work should also bring in what other related but relevant researchers have discovered to help in having a scientific analysis of the work. The tables did not help much in the analysis, it made the discussion very repetitive.

 The article should be indexed when these are taken care of.

Is the work clearly and accurately presented and does it cite the current literature?

Partly

If applicable, is the statistical analysis and its interpretation appropriate?

Partly

Are all the source data underlying the results available to ensure full reproducibility?

Yes

Is the study design appropriate and is the work technically sound?

Partly

Are the conclusions drawn adequately supported by the results?

Yes

Are sufficient details of methods and analysis provided to allow replication by others?

Partly

Reviewer Expertise:

Semantics, Pragmatics, and Discourse Analysis

I confirm that I have read this submission and believe that I have an appropriate level of expertise to confirm that it is of an acceptable scientific standard.

# Reviewer report: Reviewer response for version 1

**DOI:** 10.5256/f1000research.56826.r143696

**Published:** 2022-08-03

**Authors:** Sisanda Nkoala

**Affiliations:** 1 Media Studies, Cape Peninsula University of Technology, Cape Town, South Africa

This article states that it uses the critical discourse analysis method to analyse Indonesia's struggle for independence in newspapers published in North Sumatra in 1916-1925. The author writes very clearly, and the argument advanced is articulated well. The newspapers sampled provide depth and allow the reader to get a good overview of the content covered in the publications over the 9 year period considered. The paper’s strength is definitely the clarity with which the writer articulates themselves and the logical flow of the paper.

 A major weakness is the claim that critical discourse analysis has been used as a method in this paper. From the tables given in the results and the discussion engaged in the discussion section, the approach seems to be content analysis that draws on van Dijk’s notion of structures as aspects of analysis. The paper does not engage in a discourse analysis currently because it does not really focus on the use of words to articulate issues of power as they relate to the central idea of nationalism that is being considered. In choosing to focus on quantifying what appears and how this relates to nationalism the paper employs content analysis. Thus, in this respect, I would propose the author either changes the statement “uses critical discourse analysis” as a method and rather highlights that they have done a content analysis. Alternatively, they can consider rewriting substantial aspects of the paper and actually engaging in a discourse analysis.

 Another area that the paper could improve on is the repetitive paragraphs that are aimed at explaining what is in the table. One can assume that the tables have been presented to give a consolidated view of the contents of the papers, which they indeed do. When the author then goes ahead and writes another paragraph for each table, essentially repeating what is presented there, the paper appears repetitive at these points. The paragraphs should be used to draw the reader’s attention to specifically significant aspects of the table, which will then be ventilated further in the discussion.

 The other area of recommendation is to strengthen the discussion section. If indeed the paper is about the discourse in these papers, then the author needs to make an argument in the discussion that ventilates issues of power, as expressed in the language used in these publications, and not simply sum up what has been presented in the results. The discussion section should also bring in the voices of other scholars who have written on discourse and language in newspaper, or issues of nationalism and newspapers to demonstrate how the findings of the paper speak to existing scholarship in this respect. If, as appears to be the case currently, the paper is a content analysis paper, the author must still engage with scholarship on newspaper content and nationalism, even if it is in reference to studies from other contexts.

 I have included other comments in the paper:

 “The methods section could benefit from a discussion of CDA specifically in relation to mass media. Van Dijk has written on this, so the author could easily bring in his perspectives on the centrality of discourse on newspapers as texts and tie it into how they will speak to the manner in which notions of nationalism are articulated in these publications”.

 “While I appreciate the histographical information provided here, I do not think it adds in terms of address the issue of discourse and nationalism. If the author wishes to draw our attention to the development of indigenous newspapers in Indonisia, then the table below suffices and a summary of the most salient aspects of this, as they relate to the paper’s central argument, can be highlighted in a paragraph or at most two. The history can be mentioned only as it relates to the issue of nationalism”.

 “As with description on the contents of Table 1, I would advise the author not to spend too much writing what is in the table above, unless it is to elaborate on a specific aspect eg highlighting which one had the most, which one had the least etc.”.

Is the work clearly and accurately presented and does it cite the current literature?

Yes

If applicable, is the statistical analysis and its interpretation appropriate?

Not applicable

Are all the source data underlying the results available to ensure full reproducibility?

Yes

Is the study design appropriate and is the work technically sound?

No

Are the conclusions drawn adequately supported by the results?

Partly

Are sufficient details of methods and analysis provided to allow replication by others?

Yes

Reviewer Expertise:

media studies, discourse analysis, rhetorica studies, multlingualism

I confirm that I have read this submission and believe that I have an appropriate level of expertise to confirm that it is of an acceptable scientific standard, however I have significant reservations, as outlined above.
